# Low HER2 expression is a predictor of poor prognosis in stage I triple-negative breast cancer

**DOI:** 10.3389/fonc.2023.1157789

**Published:** 2023-03-27

**Authors:** Tomomi Sanomachi, Hitomi Sumiyoshi Okuma, Rui Kitadai, Asuka Kawachi, Shu Yazaki, Momoko Tokura, Motoko Arakaki, Ayumi Saito, Shosuke Kita, Kasumi Yamamoto, Aiko Maejima, Yuki Kojima, Tadaaki Nishikawa, Kazuki Sudo, Tatsunori Shimoi, Emi Noguchi, Yasuhiro Fujiwara, Hirokazu Sugino, Sho Shiino, Akihiko Suto, Masayuki Yoshida, Kan Yonemori

**Affiliations:** ^1^ Department of Medical Oncology, National Cancer Center Hospital, Tokyo, Japan; ^2^ Department of Diagnostic Pathology, National Cancer Center Hospital, Tokyo, Japan; ^3^ Department of Breast Surgery, National Cancer Center Hospital, Tokyo, Japan

**Keywords:** stage I triple-negative breast cancer, HER2-low, HER2-0, prognosis, prognostic factor

## Abstract

**Introduction:**

Triple-negative breast cancer (TNBC) is negative for hormone receptors and human epidermal growth factor receptor 2 (HER2). In stage I TNBC, adjuvant therapy or follow-up are performed according to risk factors, but clinical trial data is scarce. In recent years, it has been reported that HER2-low cases (1+/2+ and *in situ* hybridization negative) have different prognoses than HER2-0 cases. However, the risk of recurrence and risk factors in this HER2-low population for stage I TNBC have not yet been investigated.

**Methods:**

Herein, out of 174 patients with TNBC who underwent surgery from June 2004 to December 2009 at the National Cancer Center Hospital (Tokyo), we retrospectively examined 42 cases diagnosed as T1N0M0 TNBC after excluding those treated with preoperative chemotherapy.

**Results:**

All patients were female, the median age was 60.5 years, and 11 cases were HER2-low and 31 cases were HER2-0. The median follow-up period was 121 months. Postoperative adjuvant therapy was administered in 30 patients and recurrence occurred in 8 patients. HER2-low cases showed a significantly shorter disease-free survival (HR: 7.0; 95% CI: 1.2– 40.2; P=0.0016) and a trend towards shorter overall survival (hazard ratio [HR]: 4.2, 95% confidence interval [CI]: 0.58–31.4) compared with that of HER2-0 cases. HER2 was also identified as a factor for poor prognosis from the point- estimated values in univariate and multivariate analyses after confirming that there was no correlation between the other factors.

**Conclusion:**

For patients with stage I TNBC, the HER2-low population had a significantly worse prognosis than the HER2-0 population.

## 1 Introduction

Breast cancer is the most prevalent cancer among women worldwide with more than 2 million new cases reported in 2020 (1). In Japan, the prevalence of breast cancer is lower than that in Western countries; however, it is still the most common cancer type among Japanese women (2). Breast cancer is divided into subtypes depending on hormone receptor (estrogen receptor [ER] and progesterone receptor [PgR]) and human epidermal growth factor receptor 2 (HER2) expression. ER-, PgR-negative, HER2-0, or HER2-low-expressing triple-negative breast cancer (TNBC) account for 15-20% of all breast cancers (3). Patients with TNBC have poor chemotherapy response rates due to tumor heterogeneity and frequent development of treatment resistance, resulting in a worse prognosis compared to that in other subtypes (4, 5). Although more than 90% of breast cancers do not metastasize at the time of diagnosis, metastasis is a key reason for poor prognosis in TNBC; its 5-year survival rate is 65% for local tumors and 11% for distant metastasis, which is worse than that for other subtypes (4). As TNBC is defined as both hormone receptor-negative and HER2 negative, neither hormone therapy nor HER2 targeted therapy can be applied, and chemotherapies are the main treatments. However, immune checkpoint inhibitors are treatment options for patients with TNBC (6). Both atezolizumab and pembrolizumab were approved in 2022 in Japan for treatment of the early stages of TNBC and in the metastatic setting (7). However, perioperative adjuvant therapy and follow-up are still performed for early-stage TNBC according to the individual risk factors. Owing to TNBC heterogeneity (8), there are no precision treatments for this subtype. Additionally, clinical trial data for these early-stage patients are still scarce, which impacts the assessment of therapy effectiveness and accurate predictive markers.

However, HER2 expression may be another factor to be considered when treating early-stage TNBC. HER2 is a receptor involved in cell proliferation and is amplified or overexpressed in approximately 18–20% of primary invasive breast cancers (9). Additionally, HER2-low expression, defined as HER2 1+, HER2 2+, or HER2-*in situ* hybridization (ISH)-negative, has been shown to influence prognosis (10), and a number of HER2-low cases are now included in conventional TNBC diagnosis. The prognosis differs between HER2-low and HER2-0 cases (11), and for HER2-low cases, a clinical trial (DESTINY-Breast04) has shown that the anti-HER2 drug trastuzumab deruxtecan is highly effective in metastatic HER2-low breast cancer (12). Therefore, it is hypothesized that the risk of recurrence and therapeutic effects may differ between HER2-low and HER2-0 cases of early-stage TNBC. Despite previous reports focusing on this population (13) these included all subtypes at different stages. Thus, the recurrence risk and prognostic relationship between HER2-low/0 expression and stage I TNBC have not yet been investigated. Clarifying the relationship between low HER2 expression, recurrence risk, and prognostic factors in HER2-low stage I TNBC will enable predictions of therapeutic response and new therapeutic strategies. These results may contribute to personalized treatments for the TNBC population. Therefore, in this retrospective study, we investigated the involvement of low HER2 expression in recurrence risk and prognosis of patients with stage I TNBC.

## 2 Materials and methods

### 2.1 Patient population selection

We initially included patients who were diagnosed with TNBC and underwent surgery at the National Cancer Center Hospital (NCCH) (Tokyo, Japan) between June 2004 and December 2009 (N=174). According to UICC TNM classification 8th, stageI breast cancer is T0-1N0-1miM0. We further selected patients diagnosed with stage I TNBC, excluding those who underwent preoperative chemotherapy, and no axillary lymph node metastasis who were ER-negative, PgR-negative, and HER2-0, 1+, 2+, plus HER2-ISH negative (N=42). All 42 cases were female. Negative ER/PgR status was defined as less than 1% or 0% according to the American Society of Clinical Oncology/College of American Pathologists 2018 guidelines (14). Disease-free survival (DFS), overall survival (OS), and patient background (age, tumor size, nuclear grade, HER2/ER/PgR status, surgical procedure, administration of adjuvant radiotherapy, and adjuvant chemotherapy) were obtained from the electronic medical records. The clinical data were retrospectively evaluated, and we examined the predictors of DFS, OS, recurrence risk, and prognosis based on different HER2 expressions in stage I TNBC cases. This study was conducted in compliance with the research protocol established by the Department of Medical Oncology of NCCH. This study was approved by the ethical review board of the NCCH (No. 2014092) and conducted in full compliance with the Declaration of Helsinki.

### 2.2 Pathological examinations

Pathological examinations were performed using formalin-fixed paraffin-embedded specimens. For HER2 evaluation, specimens prepared at the surgical admission were used. The specimens were provided by the Biobank, in accordance with the National Cancer Research Center Biobank Specimen Usage Detailed Regulations. The specimens were assigned anonymized numbers and provided to two pathologists at the NCCH. The pathologists re-evaluated the specimens and determined the HER2 status according to the American Society of Clinical Oncology/College of American Pathologists 2018 guidelines. HER2 status on representative tumor cut surfaces of surgical resection specimens was examined. Sections were stained using a HercepTest II (DakoCytomation, Glostrup, Denmark), strictly following the manufacturer’s guidelines, or CB11 (BioGenex, San Ramon, CA, USA). In IHC 2+ cases, *HER2* FISH testing was performed using PathVysion (Abbott Molecular Inc., Des Plaines, Illinois, USA). For hormone receptors (ER and PgR), an Allred score of 2 or less was considered negative.

### 2.3 Clinical results and statistical analysis

After dividing stage I TNBC into HER2-low cases, defined as HER2 1+ or 2+ (ISH negative), and HER2-0 cases, we compared DFS and OS. We also examined factors that could affect DFS in HER2-low cases and HER2-0 cases; age, tumor size, surgery, adjuvant chemotherapy, adjuvant therapy, histological grade, nuclear grade, estrogen receptor, progesterone receptor, and HER2 status. Univariate analyses of continuous and nominal variables and multivariate analyses were performed using the Wald test in the Cox proportional-hazards model. The significance level of the P value was set at 0.05. OS was defined as the interval from the date of surgery to the date of any relevant event, which included death or loss to follow-up. DFS was time-limited by death, recurrence, or second cancer, whichever occurred first. For those lost to follow-up (N=37), the date of censoring was the date of final confirmation of survival. Survival analysis was performed by plotting Kaplan–Meier curves using the log-rank test. JMP 14.0.0 (SAS Institute Japan Corp. Japan) and GraphPad Prism version 8.4.3 (MDF Co., Ltd. Japan) software were used for statistical analysis.

## 3 Results

**Figure 1:**
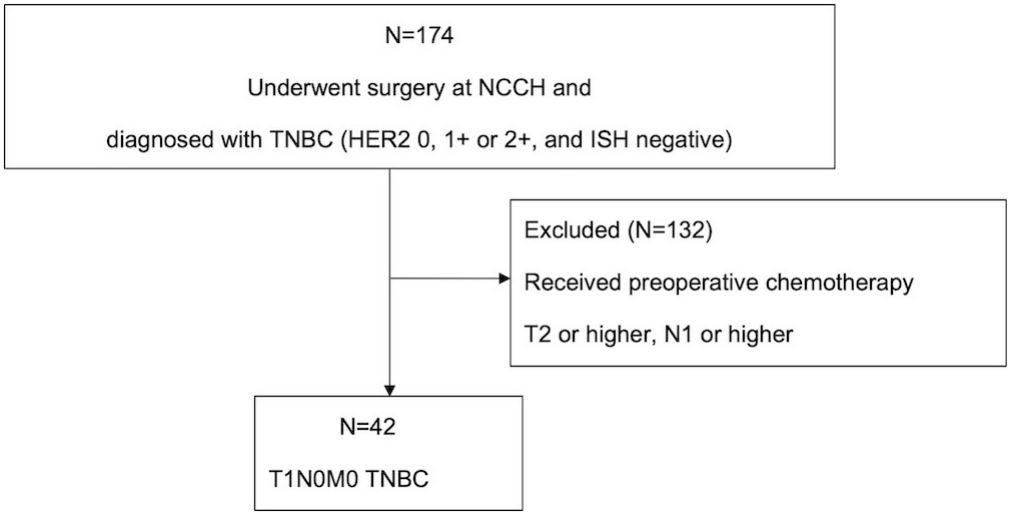
Flow diagram of the patient selection process. In total, 174 patients with TNBC were considered as candidates. Of these, 132 patients who underwent preoperative chemotherapy and those with stage T2 or higher N1 or higher were excluded from the analysis. Finally, 42 T1N0M0 TNBC were included in the analysis and followed up until October 2022. NCCH, National Cancer Center Hospital; TNBC, triple-negative breast cancer; ISH, *in situ* hybridization.

**Figure 2:**
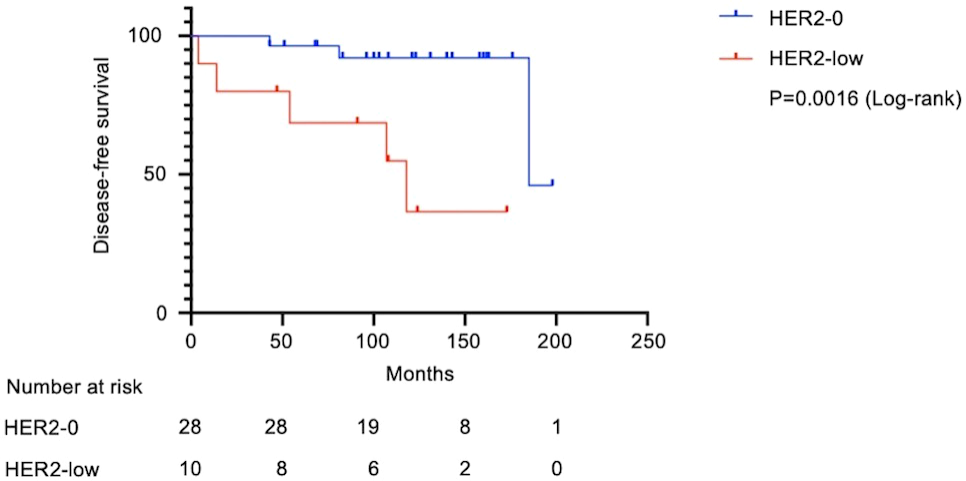
Kaplan–Meier survival curves for disease-free survival in HER2-low and HER2-0 cases. Of the 42 patients with stage I TNBC, 38 patients were analyzed after excluding 4 patients whose recurrence time was unclear in the medical records. Median survival was 118 months in the HER2-low group and 185 months in the HER2-0 group. The HER2-low group had significantly shorter DFS (Log-rank test, HR: 7.0; 95% CI: 1.2–40.2; P=0.0016). DFS, disease-free survival; HR, hazard ratio; CI, confidence interval.

**Figure 3:**
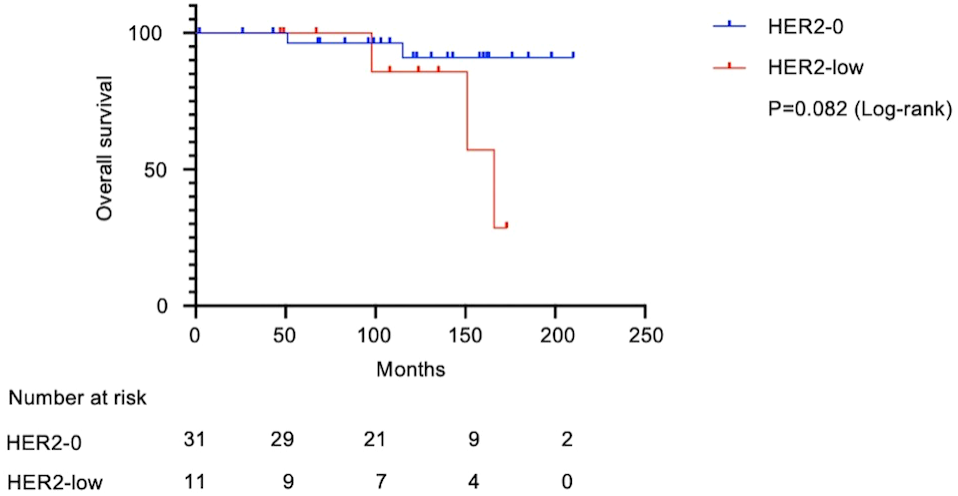
Kaplan–Meier survival curve of overall survival in HER2-low and HER2-0 cases. In total, 42 patients with Stage I TNBC were analyzed. Median survival was 166 months in the HER2-low group and undefined in the HER2-0 group. The HER2-low group had shorter OS (Log-rank test, HR: 4.2; 95% CI: 0.58–31.4; P=0.082). OS, overall survival; HR, hazard ratio; CI, confidence interval.

**Figure 1** shows the flow diagram for patient inclusion or exclusion. **Table 1** shows the baseline characteristics of the 42 patients. These patients had a median follow-up of 121 months (range: 2-210 months), a median age of 60.5 years (range: 33–84), and tumor sizes of T1a/T1b/T1c = 2/11/29. Adjuvant therapy was administered to 30 patients, including 26 (61.9%) who received chemotherapy and 12 (28.6%) who received radiotherapy. Pathological features were grade 3 in both the histological grade (HG) and nuclear grade (NG) in the majority of cases (Grade 3 HG/NG = 73.8%/76.2%), and all cases were negative for ER and PgR. Stage I TNBCs usually include a variety of histologic types, such as apocrine carcinoma, adenoid cystic carcinoma, and dysplastic carcinoma, as well as invasive ductal carcinoma of the breast. In this study, we did not arbitrarily exclude these cases, but all cases were invasive ductal carcinoma. There were 11 (26.2%) HER2-low cases and 31 (73.8%) HER2-0 cases. There were eight cases of recurrence in the patient population. A comparison of HER2-low and HER2-0 cases showed differences in the surgical procedures. HER2 1+/2+ (ISH-negative) positivity was compared between the medical records and pathologist diagnosis (**Supplementary Table 1**), and the agreement rate was higher than in previous studies (15). **Figure 2** shows the Kaplan–Meier curves for DFS in HER2-low and HER2-0 cases. The HER2-low group had a shorter median DFS than the HER2-0 group (HER2-low: 118 months; HER2-0: 185 months) (hazard ratio [HR]: 7.0; 95% confidence interval [CI]: 1.2–40.2; P=0.0016). **Figure 3** shows the Kaplan–Meier curves for OS in HER2-low and HER2-0 cases. HER2-low cases had shorter OS than HER2-0 cases (HER2-low: 166 months; HER2-0: not reached) (HR: 4.2; 95% CI: 0.58–31.4; P=0.082). Adverse events during treatment were within the known limits in all cases, and there were no treatment-related deaths. **Tables 2**, **3** shows the results of univariate and multivariate analyses of DFS. In the univariate analysis, adjuvant therapy, NG, and HER2 status were identified as prognostic factors affecting DFS. Confirming that there was no correlation between the other factors, based on the univariate and multivariate point estimates, HER2-low status was the factor contributing the most to poor patient prognosis.

**Table 1:** Baseline patient characteristics.

	Total N=42	HER2-low N=11	HER2-0 N=31
Median follow-up time Months (range)	121 (2-210)	108 (2-173)	121 (2-210)
Age Median years (range)	60.5 (33-84)	58 (43-76)	64 (33-84)
Tumor size			
T1a T1b T1c	2 (4.8) 11 (26.2) 29 (69.0)	1 (9.1) 4 (36.4) 6 (54.5)	1 (3.2) 7 (22.6) 23 (74.2)
Surgery			
Lumpectomy Mastectomy	23 (54.8) 19 (45.2)	3 (27.3) 8 (72.7)	20 (64.5) 11 (35.5)
Adjuvant chemotherapy			
Yes No	26 (61.9) 16 (38.1)	7 (63.6) 4 (36.4)	19 (61.3) 12 (38.7)
Adjuvant radiotherapy			
Yes No	12 (28.6) 30 (71.4)	1 (9.1) 10 (90.9)	11 (35.5) 20 (64.5)
Adjuvant therapy			
(chemotherapy and/or radiotherapy) Yes No	30 (71.4) 12 (28.6)	7 (63.6) 4 (36.4)	23 (74.2) 8 (25.8)
Histological grade			
1/2/3	0 (0)/ 11 (26.2)/ 31 (73.8)	0 (0)/ 2 (18.2)/ 9 (81.8)	0 (0)/ 9 (29.0)/ 22 (71.0)
Nuclear grade			
1/2/3	4 (9.5)/ 6 (14.3)/ 32 (76.2)	2 (18.2)/ 1 (9.1)/ 8 (72.7)	2 (6.5)/ 5 (16.1)/ 24 (77.4)
Estrogen receptor			
0/less than 1%	39 (92.9)/ 3 (7.1)	10 (90.9)/ 1 (9.1)	29 (93.5)/ 2 (6.5)
Progesterone receptor			
0/less than 1%	38 (90.5)/ 4 (9.5)	10 (90.9)/ 1 (9.1)	28 (90.3)/ 3 (9.7)

A total of 42 patients were included. The number of cases and the percentage of the total are shown in parentheses for each factor.

**Table 2:** Univariate analyses result for disease-free survival.

		Univariate analysis		
	N=38	Hazard ratio	95% CI	P value
Age				
≤ 60 > 60	21 17	4.6 1	0.79-87.5	0.16
Tumor size				
T1a/T1b T1c	10 28	1 0.81	0.18-5.8	0.80
Surgery				
Lumpectomy Mastectomy	22 16	1 2.3	0.50-11.6	0.29
Adjuvant chemotherapy				
Yes No	24 14	1 3.7	0.74-17.1	0.11
Adjuvant therapy				
Yes No	27 11	1 7.4	1.6-37.8	0.0096*
Histological grade				
1/2 3	9 29	1 0.54	0.13-2.7	0.41
Nuclear grade				
1/2 3	7 31	1 0.12	0.023-0.54	0.0055*
Estrogen receptor				
0 less than 1%	35 3	1 2.1	0.11-12.7	0.48
Progesterone receptor				
0 less than 1%	34 4	1 1.2	0.064-7.1	0.86
HER2				
0 1+/2+ and ISH negative	28 10	1 9.0	1.9-62.8	0.0090*

Of the 42 patients with Stage I TNBC, 38 were analyzed, excluding four patients whose recurrence time was unclear in the medical records.

The reference for the hazard ratio for each item was set at 1. Factors with significant differences are indicated by asterisks. Patients were divided into HER2-0 or HER2-low (1+/2+ and ISH negative) according to HER2 status. HR, hazard ratio; CI, confidence interval.

**Table 3:** Multivariate analyses result for disease-free survival.

		Multivariate analysis		
	N=38	Hazard ratio	95% CI	P value
Age				
≤ 60 > 60	21 17	2.97 1	0.40-60.0	0.34
Adjuvant therapy				
Yes No	27 11	1 1.3	0.076-16.7	0.84
Nuclear grade				
1/2 3	7 31	1 0.25	0.017-3.5	0.31
HER2				
0 1+/2+ and ISH negative	28 10	1 6.5	1.2-50.9	0.038*

Of the 42 patients with Stage I TNBC, 38 were analyzed, excluding four patients whose recurrence time was unclear in the medical records.

The reference for the hazard ratio for each item was set at 1. Factors with significant differences are indicated by asterisks. Patients were divided into HER2-0 or HER2-low (1+/2+ and ISH negative) according to HER2 status. HR, hazard ratio; CI, confidence interval.

## 4 Discussion

In a cohort of patients with stage I TNBC, we found that low HER2 expression was a negative prognostic factor. HER2-low expressed TNBC showed a DFS of 118 months compared to 185 months for HER2-0 TNBC.

New treatment strategies are limited for early-stage TNBC, and the prognosis remains poor compared to that of other subtypes (16). According to the National Comprehensive Cancer Network guidelines (17), adjuvant chemotherapy is recommended for TNBC tumors larger than 1 cm, but there is no consensus for smaller tumors. However, despite their size, these tumors are life-threatening. To characterize this cohort with stage I TNBC tumors, we evaluated patient DFS, OS, and background (age, tumor size, NG, HER2/ER/PgR status, surgical procedure, and whether adjuvant radiotherapy or chemotherapy was administered). Among these breast cancer-related factors, HER2 1+, 2+, and ISH negative were termed HER2-low, which has been reported to have different characteristics from the HER2-0 group (18). Therefore, we focused on HER2-low, which is present in some TNBC cases, as a breast cancer-related factor affecting recurrence risk and treatment choice for patients with TNBC. In our cohort, we identified that 26% of patients with stage I TNBC were HER2-low, consistent with the expected proportion (19, 20). Both univariate and multivariate analyses were performed for factors expected to affect the risk of recurrence, such as age (21), NG (22), and adjuvant therapy. HER2 was identified as a negative prognostic factor in both univariate and multivariate analyses of DFS in stage I TNBC. In addition, the Kaplan–Meier curves for DFS demonstrated that HER2-low cases had a statistically worse prognosis.

There are two explanations for this worse prognosis of HER2-low patients compared to that of HER2-0 patients: IHC false-negative results and molecular subtype differences.

In breast cancer, the definitions of hormone receptor and HER2 status have changed several times since 2004. In this study, we reexamined hormone receptor expression and HER2 status using the 2022 criteria and found a high concordance rate. However, we did not perform molecular analysis, such as mRNA testing, which may indicate discordance between the molecular and protein levels of these tumors. However, this molecular testing is beyond today’s clinical practice. With these limitations in mind, we believe that there is a molecular difference between HER2-low and HER2-0 subtypes. Recently, Ziteng Li et al. (23) showed that as a pan-cancer predictive biomarker for HER2-targeted therapy, the HER2 index, combined with HER2 multi-omics capabilities, can help identify potential candidates for such therapy in multiple tumor types, including breast cancer. Incorporating transcription patterns into the assessment of HER2 status is expected to lead to better patient selection, which should be taken into account for “future work”.

TNBC, an already heterogenous cancer, can be further subdivided by molecular subtypes and HER2 status using microarray assays (24). According to PAM50 analysis, approximately 60–80% of TNBC cases are basal-like, but approximately 10% are classified as HER2-enriched, which has been reported to have a worse prognosis (25). Lehmann et al. used histopathological quantification and laser capture microdissection to classify TNBC into seven subtypes, including the subtype luminal androgen receptor (LAR) (26, 27). HER2-enriched TNBC constitutes 74% of the LAR subtype (28) and the LAR subtype has been reported to have a significantly lower pathological complete response rate than that of other subtypes receiving preoperative chemotherapy (29). It has been suggested that the LAR subtype is inherently insensitive to chemotherapy. These and our results suggest that HER2-low may be a predictor of poor prognosis as a surrogate marker for the HER2-enriched subtype in TNBC.

In TNBC, the recurrence risk peaks within 3 years after diagnosis, and recurrence events associated with poor prognosis are more common within 5 years. Additionally, mortality increases 5 years after diagnosis (30). In this study, a statistically significant difference was observed in DFS between HER2-low and HER2-0 cases by the log-rank test, but the difference in OS was not statistically significant. This outcome is thought to be because appropriate post-treatment reduced the risk of death. In breast cancer, cases of late recurrence are often encountered. For this reason, we extended the analysis period to more than 10 years, making it more reliable.

This study has three limitations. First, the HER2 mRNA level and the IHC/FISH results in HER2-0 cases were not compared, and we cannot rule out false-negative test results. Second, the number of cases was limited because this study was conducted at a single institution. Additionally, there were few recurrent events, and the univariate and multivariate models were not highly stable. The small sample size of this study hinders additional statistical models or adjustments. In univariate analysis, for DFS, adjuvant therapy (either or both chemotherapy and radiation therapy) was a significant prognostic factor, whereas adjuvant chemotherapy alone was not. According to NCCN guideline, in T1-3, N0-1, M0 breast cancer, radiation therapy is mandatory for partial mastectomy, total mastectomy should be treated with radiation therapy according to the needs of the individual case. The present study included 22 cases of Lumpectomy and 16 cases of Mastectomy, which may have been influenced by the presence of a certain percentage of cases requiring additional adjuvant radiation therapy in addition to adjuvant chemotherapy. In this study, the median age of the target patients was 60.5 years old, and 16 patients (38.1%) aged 65 years or older were included. In a previous report, 35% of patients older than 65 years with TNBC were HER2-enriched, and age may be an important predictor in larger patient population sizes (31). Considering our limited sample size, previous reports (32, 33) showed that HER2-low cases had a better prognosis than HER2-0 cases, a result different from our own. However, none of these reports included hormone positivity/negativity- or stage-specific factors. Denkert et al. (33) and de Moura et al. (34) included cases of preoperative adjuvant therapy, which were excluded in our study. Our study focused only on stage I TNBCs, which may have led to different results because of the purity of the population. Another factor may be the discrepancy in the HER2-low expression degree in TNBC between early- and advanced-stage samples (35). Tarantino et al. reported that as breast cancers develop into advanced stages, in most part, they have shifts from HER2-0 to HER2-low; the vice versa being less frequent. This result suggests that previous clinical trials that were not stage-specific and/or included a large number of advanced-stage cases may have had an expanded HER2-low population, leading to different results. However, HER2-low expression instability in early-stage TNBC remains unclear. Although several reports related to HER2-low breast cancer prognosis differ from ours, a recently published report supports our results (36). Di Cosimo et al. found that although HER2-low cases tended to have a worse prognosis than HER2-0 cases, there was no difference in DFS. As in our study, the HER2 status of the included cases was determined by the American Society of Clinical Oncology/College of American Pathologists 2018 criteria rather than extracted from medical records, as has been done in previous reports. However, this study only included breast tumors ≥ 2 cm (T2 or larger), which does not clarify the role of HER2-low as a prognostic factor in stage I TNBC. Third, since this was a retrospective study with a small number of cases, verification in prospective clinical trials is necessary.

Recent clinical trials related to HER2-low include the NSABP B-47/NRG and DESTINY-Breast04 trials and the drugs used in these trials have been approved by the FDA. The NSABP B-47/NRG trial is a randomized phase III study of the add-on effect of trastuzumab, a humanized HER2 monoclonal antibody, on two standard regimens for HER2-low, high-risk, primary invasive breast cancers. This treatment change showed no significantly increase in invasive DFS or OS in the subgroup analysis classified by hormone receptor expression level (13). However, hormone-positive breast cancer accounted for 82.7% of all patients, and this trial was not stage-specific. The results of the DESTINY-Breast04 trial were published in 2022, which was an international multicenter joint phase 3 study targeting patients with HER2-low metastatic/recurrent breast cancer who had received pretreatment with chemotherapy (12). In this study, trastuzumab deruxtecan, which is trastuzumab conjugated to a camptothecin derivative with topoisomerase I inhibitory effects, was used as the tested drug. Encouragingly, it significantly prolonged PFS, which was the primary endpoint; however, this study included hormone receptor-positive, unresectable, metastatic, and recurrent HER2-low breast cancers. The effect of DS-8201a/trastuzumab deruxtecan on early HER2-low breast cancer and TNBC (HER2-low) is currently under investigation. However, based on the results of this study, we expect that this drug will be effective for stage I TNBC with low HER2 expression. According to a previous report by Tolaney et al., reported in a single-group, uncontrolled trial examining the efficacy of adjuvant paclitaxel and trastuzumab for stage T1-2 HER2- positive breast cancer (37). In this study, 91% of patients with T1mic-c cancers were included, indicating that this combination therapy will be useful for stage I HER2-positive breast cancer. The patient outcomes exceeded the 3-year survival rate for patients without invasive disease (37). In this study, 91% of patients with T1a-c cancers were included, indicating that this combination therapy will be useful for stage I HER2-positive breast cancer. However, the effect remains unclear as more than half of these cases were hormone receptor-positive, and HER2 status was not classified as HER2-low or -0. Therefore, it is desirable to further investigate postoperative adjuvant therapy, including anti-HER2 therapeutic agents, in preventing recurrence in patients with stage I TNBC with low HER2 expression.

In conclusion, among stage I TNBC cases, HER2-low cases had a significantly shorter DFS and a trend towards shorter OS compared to HER2-0 cases. HER2 was also identified as a factor for most poor prognosis from the point-estimated values in univariate and multivariate analyses after confirming that there was no correlation between the other factors. HER2-low may be a predictor of poor prognosis as a surrogate marker for the HER2-enriched subtype in stage I TNBC cases.

## Data availability statement

The raw data supporting the conclusions of this article will be made available by the authors, without undue reservation.

## Ethics statement

The studies involving human participants were reviewed and approved by the Ethics Committee of the NCCH (No. 2014092). Written informed consent for participation was not required for this study in accordance with the national legislation and the institutional requirements.

## Author contributions

All authors contributed to the study conception and design. Material preparation, data collection and analysis were performed by TS and HO. The first draft of the manuscript was written by TS and all authors commented on previous versions of the manuscript. All authors read and approved the final manuscript. All authors contributed to the article and approved the submitted version.
