# Supplementary material for: Low HER2 expression is a predictor of poor prognosis in stage I triple-negative breast cancer
Source: Front Oncol. 2023 Mar 27;13:1157789. doi: 10.3389/fonc.2023.1157789 (PMC10083471; doi:10.3389/fonc.2023.1157789)
Supplement: Supplementary file 1 [file Table_1.pdf]

Supplementary Material

Low HER2 expression is a predictor of poor prognosis in stage I triple-negative breast cancer

Tomomi Sanomachi[1], Hitomi Sumiyoshi Okuma[1], Rui Kitadai[1], Asuka Kawachi[1], Shu Yazaki[1], Momoko Tokura[1], Motoko Arakaki[1], Ayumi Saito[1], Shosuke Kita[1], Kasumi Yamamoto[1], Aiko Maejima[1], Yuki Kojima[1], Tadaaki Nishikawa[1], Kazuki Sudo[1], Tatsunori Shimoi[1], Emi Noguchi[1], Yasuhiro Fujiwara[1], Hirokazu Sugino[2], Sho Shiino[3], Akihiko Suto[3], Masayuki Yoshida[2], Kan Yonemori[1]

*** Correspondence:** Hitomi Sumiyoshi Okuma MD, PhD: hsumiyos@ncc.go.jp

1 Supplementary Tables

Supplementary Table 1. HER2 status of 42 cases. Two pathologists reviewed HER2 status using the ASCO/CAP2018 criteria. The agreement between the final HER2 status and medical records was 39/42 cases (92.9%). 10 cases were stained with CB11 and 32 cases with HercepTest II.

Patient number	At or before diagnosis (ASCO/CAP2007)	Pathologist 1 At diagnosis (ASCO/CAP2018)	Pathologist 2 At diagnosis (ASCO/CAP2018)	Consensus between pathologists 1 & 2
1	0	0	0	0
2	0	0	0	0
3	0	0	0	0
4	0	0	0	0
5	0	0	0	0
6	0	0	0	0
7	0	0	0	0

8	0	1+	0 or 1+	1+
9	0	0	0	0
10	0	0	0	0
11	0	0	0	0
12	0	0	0	0
13	1+	1+	1+	1+
14	0	0	0	0
15	0	0	0	0
16	0	0	0	0
17	0	0	0	0
18	0	0	0	0
19	0	0	0	0
20	0	0	0	0
21	0	0	0	0
22	0	0	0	0
23	0	0	0	0
24	0	0	0	0
25	0	0	0	0
26	0	0	0	0
27	0	0	0	0

28	0	0	0	0
29	0 (intraductal 1+)	0	0	0
30	1+	1+	1+	1+
31	1+	2+	2+	2+/ISH negative
32	0	0	0	0
33	1+	1+	1+	1+
34	1+	1+	1+	1+
35	1+	1+	1+	1+
36	1+	1+	1+	1+
37	1+	1+	1+	1+
38	1+	1+	1+	1+
39	0	0	0	0
40	0	0	0	0
41	1+	1+	1+	1+
42	1+	0	0	0
